# Vocal Learning Versus Speech Evolution: Untangling a False Equivalence

**DOI:** 10.1002/ece3.71241

**Published:** 2025-04-03

**Authors:** Adriano R. Lameira

**Affiliations:** ^1^ ApeTank, Department of Psychology University of Warwick Coventry UK

## Abstract

The evolution of speech remains one of the most profound and unresolved questions in science. Despite significant advancements in comparative research, key assumptions about the evolutionary precursors of speech continue to be accepted with minimal scrutiny. One such assumption is the widely held belief that vocal learning—the ability to imitate and modify vocalizations—was an obligatory precondition for speech evolution. However, by the time ape‐like human ancestors emerged amid Miocene's forests, the ancestors of vocal learning species already walked the Earth and flew the skies. A head‐start of millions of years of vocal evolution didn't produce linguistic elephants, bats, or birds, suggesting that hominids' humble vocal beginnings were determinant for verbal evolution. Current evidence on extant great ape calls provides new details and insight into the extinct vocal forms and functions that allowed human ancestors to jump‐start speech evolution. By reconsidering the evolutionary processes that led to speech, this paper advocates for a shift in focus toward the hominid biotope, body, brain, and behavior, rather than treating speech as the pinnacle endpoint of vocal learning evolution and drawing misleading parallels with far‐related vocal learners.

## Abstract 2

Current evidence on extant great ape calls provides new details and expanding insights into the extinct vocal forms and functions that allowed human ancestors to jump‐start speech evolution.

The origin of speech has mystified scholars for millennia and tantalized scientists for more than a century, provocatively suggested by some to represent the hardest problem in science (Christiansen and Kirby 2003a). Because sound instantly biodegrades and does not leave a direct trace on the fossil record, the origin of speech has proven one of the most elusive among human defining traits. The ancient sounds produced by human ancestors will never be unearthed from an archaeological dig or decoded from ancient DNA. Therefore, the study of speech evolution requires a comparative approach to animal vocal behaviors that can offer extant proxies to extinct signals.

Over the last decades, the vocal (production) learning hypothesis has emerged as the dominant framework to explain speech evolution from a comparative perspective (Janik and Slater 1997; Christiansen and Kirby 2003b; Bolhuis and Wynne 2009; Fitch and Jams 2013; Jarvis 2019; Vernes et al. 2021; Wirthlin et al. 2024). It proposes that vocal learning—the capacity to expand one's call repertoire through the imitation of new sounds or modification of pre‐existing calls through acoustic feedback—was a precondition for speech evolution. As a result, far‐related species that have independently developed vocal learning have been championed as privileged models for the study of speech evolution. On land and in flight, these species include elephants, bats, songbirds, parrots, and hummingbirds.

All languages' sounds must be acquired through vocal learning by every new speaker, therefore, the vocal learning hypothesis appears self‐explanatory and has been predominantly upheld with minimal scrutiny. Yet, it suffers from critical shortcomings (Lameira 2017; Martins and Boeckx 2020; Lameira et al. 2022; Ekström, Gannon, et al. 2024), warranting a logical audit. For example, whilst learned, each individual language only makes use of a relatively trivial number of speech sounds (between ~22 and ~42, depending on the language family), with many languages across continents exhibiting full functionality with *fewer* sounds than great ape repertoires (Lameira and Moran 2025), which the vocal learning hypothesis presumes to be innate. If language can make do with fewer sounds than great apes (and other primates), whether the sounds of the first language(s) had to be obligatorily socially learned becomes theoretically questionable. Humans can build a language with a handful of sounds, but animal species capable of expanding their sound repertoire through vocal learning with tens or hundreds of new call types cannot. One is forced to ponder whether the first language(s) in the human lineage depended on the nature, rather than number, of the sounds necessary for its expression.

Intriguingly, the vocal learning hypothesis also rejects (Janik and Slater 1997; Christiansen and Kirby 2003b; Bolhuis and Wynne 2009; Fitch and Jams 2013; Jarvis 2019; Vernes et al. 2021; Wirthlin et al. 2024) any studies evincing vocal learning in great apes, which now surpass in number and scale (reviewed below) those readily accepted to prove vocal learning in other taxa (e.g., elephants, marmosets). The hypothesis also dismisses that, in humans, vowels virtually always take the form of voiced utterances, whereas voiceless utterances take the form of consonants (Lameira 2022), therefore, dismissing the direct homology in articulation and acoustics between great ape consonant‐like and vowel‐like calls and their human counterparts. Doing so, the hypothesis takes no notice or concern for explaining how vocal learning would have arisen in the Hominid family in the first place, nor the origin of consonantal and vocalic sounds. An evolutionary hypothesis that rejects the existence of, and refuses to consider, behaviors that may have been precursors to the capacity it seeks to explain defeats its own purpose. The hypothesis also misrepresents and mis‐predicts vocal learning mechanisms and capacities across other taxa (Martins and Boeckx 2020). These and other arguments are extensively covered and explained elsewhere (Lameira 2017; Martins and Boeckx 2020; Lameira and Moran 2025). Here, a new, simple thought experiment is carried out to show that there is no phylogenetic indication to conclude that speech is the endpoint of vocal learning evolution or that vocal learning was a precondition to the first language(s) in the human lineage.

## 1 Fair Equality of Evolutionary Opportunity

Although sound does not fossilize, the archaeological record confirms that the ancestral lineages of aquatic, terrestrial, and flying vocal learners were already established when the first hominids emerged within the primate order. The ancestral lineages of terrestrial and flying vocal learning species even inhabited some of the same regions as ancient hominids by the time the latter came onto the scene. Accordingly, vocal learning ancestral lineages survived and adapted to the same dramatic climate changes that swept Eurasia and Africa during the Mid (15–11 million years ago) and Late Miocene (11–5 mya) and that provided the *de facto* ground zero for speech and language origin.

This period of climatic turnover remains an ecological black box on the evolutionary timeline of spoken language, obscuring precursors and pressures. On one end, an ancestral ape‐like hominid with none or unassuming vocal capacities entered this black box, and millions of years later, came out on the other end as a verbal human. The ancestors of elephants, bats, and vocal learning birds, having gone through the same period and ecological processes, did not produce linguistic elephants, bats, or birds.

One of two scenarios transpired, hence. First, the ancestors of elephants, bats, songbirds, parrots, and/or hummingbirds were non‐vocal learners (as presumed for hominid ancestors) and developed vocal learning capacities while the hominid lineage developed speech. For example, this happened with neotropical branches of the primate order, such as marmoset monkeys (Takahashi et al. 2015; Gultekin et al. 2021), who independently developed vocal learning alongside hominid evolution one ocean away. If this scenario is correct, some non‐vocal learners became vocal learners, while other non‐vocal learners became verbal. If different outcomes were reached by different and independent lineages, then the evolution of vocal learning can only be assumed to have represented a parallel, not sequential, process from that of speech.

In the second scenario, the ancestors of elephants, bats, songbirds, parrots, and/or hummingbirds were already vocal learners. Proboscideans emerged 60 mya, bat ancestors likely capable of primitive echolocation emerged 50 mya, songbirds 50 mya, parrots 59 mya, and hummingbirds 22 mya. If speech evolved in the human lineage after it diverged from other great ape lineages, some 5–7 mya, then all vocal learning ancestors had a head start of many times the same period for evolving speech, but without producing the same results. If this scenario is correct, vocal learners remained vocal learners and non‐vocal learners became verbal.

The objective here is not to defend the likelihood of one scenario over the other, but to demonstrate that, whichever scenario we turn to, vocal learning cannot be accepted, on logical grounds, to have been an obligatory precondition for speech evolution, as hitherto advocated. No directional or causal relationship can be drawn. Lineages across taxa can evolve at distinct rates, but if the hominid clade produced qualitatively different outcomes in a time window shorter by one order of magnitude, one can only further doubt whether (in addition to the other theoretical and empirical defects of the hypothesis) comparing the trajectories of vocal learning versus speech evolution is accurate, germane, or useful.

Regardless of which evolutionary scenario ultimately proves most likely, the last 15 my operated as a great filter for speech evolution that several candidate lineages of terrestrial mammals and birds tried to transverse, but that only one succeeded in crossing—hominids. Why was this filter permeable to hominids and not the members of vocal learning lineages? To understand the evolution of speech and of minimal viable sound systems for the expression of language, the reasons why (despite contemporary and sympatric lineages with otherwise promising potential) only the call repertoire of ape‐like ancestors took a turn toward speech must be considered. We must focus on the vocal units and capacities of extant great apes, however humble these beginnings may have been.

## 2 How Did the Tortoise Cross the Speech Line Before the Vocal Learning Hares?

Modern humans derive from the same hominid stock as modern (nonhuman) great apes—orangutans, gorillas, chimpanzees, and bonobos—humans' closest living relatives. Great apes provide the most realistic living models of ancestral hominids' vocal capacities, who were yet to acquire speech. What can great apes “say” about human ancestral vocal capacities?

Similarly to each and every world's spoken languages, great ape vocal repertoires are composed by voiced vowel‐like (Ekström, Nellissen, et al. 2024) and voiceless consonant‐like calls (Lameira et al. 2014), which great apes can articulate to mimic human syllables and words (Ekström, Gannon, et al. 2024), imitate human sounds (Lameira et al. 2013; Wich et al. 2009), reproduce speech‐like rhythm (Lameira et al. 2015; Pereira et al. 2020) and combine according to various (Girard‐Buttoz et al. 2022) and complex language‐like rules (Kershenbaum et al. 2014; Lameira et al. 2024). These call combinations are then used as a canvas for advanced cognitive and language‐like functions, allowing great apes' sophisticated cognition to spill over onto the vocal domain. For example, great apes vocalize intentionally (Gruber and Zuberbuhler 2013; Schel et al. 2013), inform ignorant individuals about missing information (Crockford et al. 2017), vocally deceive others (Hardus et al. 2009), and communicate about past (Lameira and Call 2018) and future events (van Schaik et al. 2013). Great apes exert voluntary control over the movement of the lips (Ekström, Nellissen, et al. 2024), tongue, jaw (Lameira et al. 2013), and larynx (Lameira et al. 2016; Lameira and Shumaker 2019) to the extent that they can produce vowel‐like and consonant‐like calls simultaneously, a feat only matched among primates by human beatboxers (Lameira and Hardus 2023).

Like speech sounds, several great ape calls are not universal to the species but represent instead local‐specific traditions (Wich et al. 2012; Taglialatela et al. 2012; Russell et al. 2013). An example is synonym calls that vary geographically in a dialect‐like fashion, similar to “pants” and “trousers” between American and British English. For example, in one population an orangutan mother may summon her infant using a vowel‐like call; in another population, mothers may use a consonant‐like call, and yet other populations of the same species, mothers will use no call for the same context or function (Wich et al. 2012). This geographic patchwork pattern is extremely difficult to explain on the basis of genetic or ecological drift alone (Wich et al. 2012). Calls may also vary between populations in an accent‐like fashion (Watson et al. 2015; Lameira et al. 2017), similar to “tomatoes” pronounced in American and British English, and the degree of social contact experienced by individuals in the wild during development affects vocal repertoire breadth across different populations and how vocally innovative individuals are (Lameira et al. 2022).

## 3 The Ape‐Human Vocal‐Verbal Continuum: A Bottom‐Up Comparative Approach

Great ape vocal biology and behavior is clearly aligned with speech and languages in unique ways that confirm an ape‐human vocal‐verbal continuum according to shared ancestry and descent with modification, as predicted by Darwinian principles of evolution by natural evolution. The vocal learning hypothesis has been instrumental in driving comparative research over the last 25 years (Fitch 2000), but it has now proved logically flawed and insufficient to advance new insights about the evolution of speech and language in the human lineage, requiring serious reform, if not abandonment. With the exception of variation among birds (Audet et al. 2023), the hypothesis has provided no predictive or explanatory power for the phylogenetic distribution of vocal learning across mammals. Convergent evolution and analogy between far‐related taxa are theoretically considered to allow inference about selective pressures that favor the evolution of certain traits, in this case vocal learning. Yet paradoxically, in a quarter of a century, no inference has been drawn based on whale, elephant, or bird data about the selective pressures that might have favored vocal learning in hominids. This is perhaps not surprising. Whales vocally learn through their blowholes (Ridgway et al. 2012) or modified larynxes (Elemans et al. 2024), elephants through their trunks (Stoeger et al. 2012), and birds through their syrinxes, an organ that contains up to three sound sources (Garcia et al. 2017), each functionally analogous to a set of vocal folds in the mammalian larynx. Whales are aquatic and weigh up to 30 tons, elephants graze and weigh 3000 kg, songbirds fly and weigh less than 13 g.

Furthermore, inferring selective pressures through convergent evolution is a vacuous exercise if one does not know, consider, or specify the traits targeted by those pressures, and the vocal learning hypothesis rejects any insight in this regard from great ape vocal research. What exactly can be extrapolated from accepted vocal learners to ancient hominids remains paradoxically vague and idle. If we are to decipher speech and language evolution, it is now paramount to directly tackle the question of why the hominid biotope, body, brain, and behavior carved a path toward the first language(s) in a way and evolutionary tempo that no vocal learning lineage did.

The present thought experiment and the logical audit to the vocal learning hypothesis show that the provenience of the sounds used to build the first language(s) in the human lineage—vocally learned versus innate—was not as determinant as presumed so far. Socially learned or not, ape‐like ancestors already had sufficient sounds at their disposal to build a minimally viable sound system for language. Indeed, many modern languages are fully operational with fewer sounds than those used by great apes. Similar to extant great apes, ancient ape‐like ancestors probably exercised a moderate degree of vocal learning. Thus, it is reasonable to accept (at least as working null hypothesis) that some of the sounds of the first language(s) were socially sourced and others were not.

A simple, yet compelling conjecture is that the first language(s) did not rely on open‐ended repertoires or a vast array of sounds, but rather on a structured mix of vowel‐like and consonant‐like calls. Languages are combinatorically open‐ended, not acoustically open‐ended. Indeed, the two fundamental sound categories that universally combine to generate the world's languages are also present in great apes (Lameira 2017).

While this view requires future testing, it already raises new questions with a level of detail that the vocal learning hypothesis has failed to generate in decades of research. Without this level of specificity and curiosity about the vocal prerequisites for language expression in ancestral hominids, science will remain unable to tackle this central puzzle of what it means to be human. While insights from convergent evolution and analogy may still prove valuable in the future, they can only be meaningfully applied after a bottom‐up approach is undertaken and a modern theory of speech and language evolution is built and framed in light of the homologies between what great apes do vocally and what humans do verbally.

## Author Contributions

**Adriano R. Lameira:** conceptualization (lead), funding acquisition (lead), project administration (lead), validation (lead), writing – original draft (lead), writing – review and editing (lead).

## Conflicts of Interest

The author declares no conflicts of interest.

## Data Availability

The author has nothing to report.
